# Traditional Chinese medicine for stable angina pectoris via TCM pattern differentiation and TCM mechanism: study protocol of a randomized controlled trial

**DOI:** 10.1186/1745-6215-15-422

**Published:** 2014-10-30

**Authors:** Zhe Zhang, Fan Zhang, Yang Wang, Yi Du, Huiyong Zhang, Dezhao Kong, Yue Liu, Guanlin Yang

**Affiliations:** Affiliated Hospital of Liaoning University of Traditional Chinese Medicine, Shenyang, Liaoning China; Liaoning University of Traditional Chinese Medicine, 79, Chong Shan Dong Lu Road, Huanggu District, Shenyang, 110032 Liaoning China

## Abstract

**Background:**

Stable angina pectoris is experienced as trans-sternal or retro-sternal pressure or pain that may radiate to the left arm, neck or back. Although available evidence relating to its effectiveness and mechanism are weak, traditional Chinese medicine is used as an alternative therapy for stable angina pectoris. We report a protocol of a randomized controlled trial using traditional Chinese medicine to investigate the effectiveness, mechanism and safety for patients with stable angina pectoris.

**Methods/Design:**

This is a north-east Chinese, multi-center, multi-blinded, placebo-controlled and superiority randomized trail. A total of 240 patients with stable angina pectoris will be randomly assigned to three groups: two treatment groups and a control group. The treatment groups will receive Chinese herbal medicine consisting of *Yi-Qi-Jian-Pi* and *Qu-Tan-Hua-Zhuo* granule and *Yi-Qi-Jian-Pi* and *Qu-Tan-Hua-Yu* granule, respectively, and conventional medicine. The control group will receive placebo medicine in addition to conventional medicine. All 3 groups will undergo a 12-week treatment and 2-week follow-up. Four visits in sum will be scheduled for each subject: 1 visit each in week 0, week 4, week 12 and week 14. The primary outcomes include: the frequency of angina pectoris attack; the dosage of nitroglycerin; body limited dimension of Seattle Angina Questionnaire. The secondary outcomes include: except for the body limited dimension of SAQ, traditional Chinese medicine pattern questionnaire and so on. Therapeutic mechanism outcomes, safety outcomes and endpoint outcomes will be also assessed.

**Discussion:**

The primary aim of this trial is to develop a standard protocol to utilize high-quality EBM evidence for assessing the effectiveness and safety of SAP via TCM pattern differentiation as well as exploring the efficacy mechanism and regulation with the molecular biology and systems biology.

**Trial registration:**

Clinical Trials Registration: ChiCTR-TRC-13003608, registered 18 June 2013.

**Electronic supplementary material:**

The online version of this article (doi:10.1186/1745-6215-15-422) contains supplementary material, which is available to authorized users.

## Background

Stable angina pectoris (SAP) due to coronary heart disease (CHD) is defined as a major clinical manifestation of trans-sternal or retro-sternal pressure or a choking sensation or pain, which is caused by coronary insufficiency, myocardial ischemia, and hypoxia on the basis of an atherosclerotic coronary vascular lumen [1, 2]. According to World Health Organization (WHO), CHD is one of the major causes of death and has drawn the attention of traditional Chinese medicine (TCM) and western medicine (WM) researchers. In China, the prevalence and incidence of SAP are 2.4% in males and 3.2% in females, which is a serious social problem in view of its large population basis [3]. Its treatment is mainly relief of symptoms with anti-anginal medications or revascularization procedures. The anti-anginal medications are selected from three groups of anti-anginal chemical drugs including β blockers (BBs), calcium channel blockers (CCBs) and nitrates [4]. In China, a large majority of SAP patients resort to Chinese herbal medicine, acupuncture and other TCM therapies [5].

As for SAP, phlegm has become the popular trend of TCM pattern elements. The relationship between spleen deficiency generating phlegm and SAP has been recorded in the *Inner Canon of the Yellow Emperor* (*Huangdi Neijing*). Correspondingly, phlegm and spleen deficient is one of the main TCM patterns of SAP in terms of recent studies [6, 7].

The TCM pattern is a characteristic profile of clinical signs and symptoms manifested by a group of patients [4]. Wang Yong-yan, Academician of Chinese Academy of Engineering, postulated that TCM pattern had the performance characteristics that included reality inside and virtual outside, spatiotemporal dynamic, multi-dimensional interface [8] . Lu Guang-xin, TCM master, announced that the TCM pattern was the core concept and logical starting point of pattern differentiation, which contained the property of diagnostics, pathophysiology, and therapeutics [9, 10]. Hence, we may believe that TCM pattern is a physiological regulation network, which contains a regulation center, overall effective target, material basis and functional unit. Its mechanism needs to be indicated by means of TCM pattern prevention and treatment characteristics, multiple organ integration effectiveness, and functional unit network so as to guide clinical practice as well as for inspiring basic research.

Systems biology, defined as multiple comics integration and consolidation from genes, cells, the organization of individuals, is the TCM pattern efficacy and mechanism research method fitting with TCM theory, characteristics and discipline [11–13]. Hence, we utilize high throughput screening (HTS) technology of systems biology; that is transcriptomics, metabonomics, proteomics, and so on, to promote fundamental research of TCM pattern, global Chinese compound medicine selection, evaluation and technology platform. In recent years, a comprehensive approach has shown the TCM efficacy mechanism of SAP in the view of regulating lipid metabolism abnormality and resisting inflammatory expression and endothelial dysfunction [14]. However, there is not adequate understanding of the interaction and relationship between spleen deficiency generating phlegm of SAP (response system) and regulation from spleen (intervention system) through the scattered and small-scale reduction biological research. Notably, we should explore the clinical evaluation and efficacy mechanism and regulation on the foundation of systems biology and evidence-based medicine (EBM).

In this trial, we intend to investigate the efficacy mechanism for SAP with spleen deficiency and phlegm pattern through brain-gut axis regulation of lipid metabolism, immune inflammation and vascular endothelial network. Meanwhile, we attempt to further validate the effectiveness and safety in light of clinical efficacy evaluation index, which is the frequency and duration of angina pectoris attacks, health-related quality of life, and so on.

## Methods/Design

### Study design

This is a north-east Chinese, multi-center, multi-blinded, placebo-controlled and superiority randomized trail with three parallel groups. The study protocol was based on strict and scientific recommendations in the Consolidated Standards of Reporting Trials (CONSORT) statement [15] and SPIRIT 2013 Statement [16]. This trial was supported financially by the National Basic Research Program of China ‘973 Program’ (Number 2013CB531704). The trial was registered in Chinese ClinicalTrials.gov with approval number ChiCTR-TRC-13003608.

**Figure 1:**
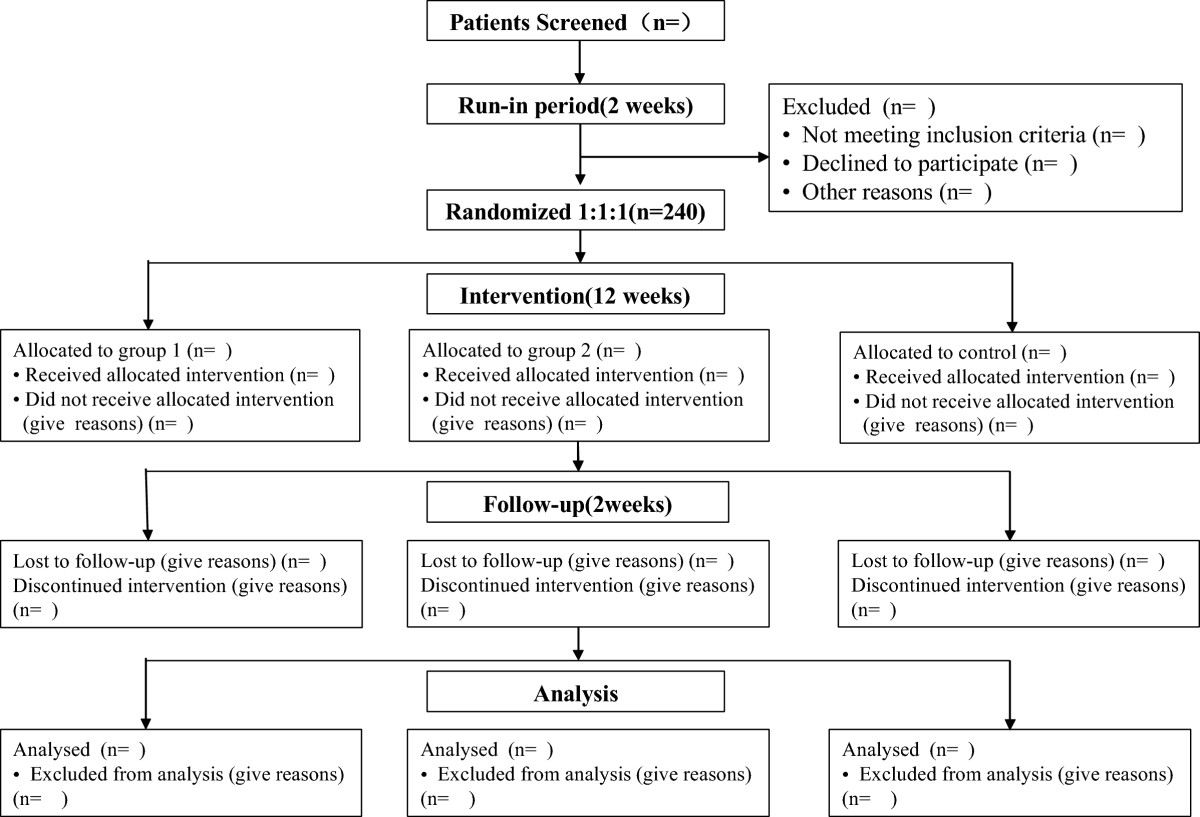
**Trial flow chart.**

Two hundred and forty participants will be recruited at six large comprehensive hospitals in China, which are: Affiliated Hospital of Liaoning University of TCM, Liaoning Institute of Chinese Medicine, Affiliated Hospital of Inner Mongolia National University, Shen-Zhou Hospital of Shenyang Medical College, First Affiliated Hospital of China Medical University, and Sheng-Jing Hospital of China Medical University. After a two-week run-in period, eligible subjects will be randomly assigned to one of 3 groups, *Yi-Qi-Jian-Pi* and *Qu-Tan-Hua-Zhuo* group (Group 1), *Yi-Qi-Jian-Pi* and Qu-Tan-Hua-Yu group (Group 2), and the placebo group. All three groups then underwent a 12-week treatment and a 2-week follow-up period. The flow chart is listed in Figure 1.

### Randomization and allocation concealment

A total of 240 patients will be randomly assigned in a ratio of 1:1:1 using the statistical package. To guarantee rigorous methodology, stratified block randomization and central coordination will be initiated. The unique code will be assigned to each newly eligible participant and properly preserved in the trial management board. This procedure ensures each eligible patient having an equal probability of being assigned to three different groups, which will not be influenced by the researchers. A statistician expert of the Beijing Bozhiyin Science and Technology Co. Ltd, Beijing, China will act as the coder and they will be shielded from patient recruitment.

### Blinding

This trial is a multi-blinded design and researchers, participants, drug management staff, data collection staff and statisticians will be blinded during the trial period. When an emergency occurs (such as a serious adverse events), or doctors must know the treatment of patients who require intervention, the researcher should immediately inform the main researcher to unblind the central random system. Moreover, the main researchers and trial monitors will be informed immediately. Once the blind letter is open following an emergency, the participant will be eliminated from the trial and the researcher should note the reason, blind time and place of uncovering in the Case Report Forms (CRFs) together with the main researcher, Institutional Review Board (IRB) and staff who perform the unblinding.

### Participants

The study will enroll 240 patients, which fulfill the following items of criteria in this trail.

### Diagnostic criteria

1. WM diagnostic criteria of diagnosis and treatment guidelines for chronic stable angina pectoris according to the Chinese Society of Cardiology in 2007 [17]
2. TCM diagnostic criteria of Chinese Internal Medicine according to the 11th Five-Year national programmed textbooks together with expert demonstration. The signs and symptoms as follows: chest distress superior and pain inferior, excessive phlegm, shortness of breath, heavy limbs, obesity, attack or aggravation on a rainy day, exhaustion, loss of appetite, loose stool, expectoration, plump tongue and side tooth marks, greasy and glossy coating, slippery pulse

### Inclusion criteria

1. Diagnosis of CHD with SAP as well as spleen deficiency and phlegm pattern according to WM and TCM
2. Age: male: 45 to 75 years old (including 45 and 75 years old); female: 50 to 75 years old (including 50 and 75 years old)
3. Main coronary artery stenosis between 50% and 75% or branch coronary artery stenosis between 50% and 100% in the previous 3 months as shown on coronary angiogram or coronary computed tomography (CT) examination report
4. Patients meet Class II to IV of the Canadian Cardiovascular Society (CCS) angina pectoris severity grade
5. Able to understand and sign a written informed consent

### Exclusion criteria

1. Pattern differentiation except for spleen deficiency and phlegm pattern
2. A history of chest pain caused by the unstable angina pectoris (UAP), hypertrophic cardiomyopathy (HCM), neurosis, post-menopausal pattern, gastric and esophageal reflux disease (GERD), and so on
3. Main coronary artery (right coronary artery (RCA), left main stem (LM), left anterior descending (LAD), left circumflex (LCX)) more than 75% stenosis according to coronary angiogram or coronary CT
4. Acute myocardial infarction in the previous half year or completely revascularization history within a year
5. Severe heart failure (class IV of heart function), malignant arrhythmia, hypertension higher than 160/100 mmHg within standard anti-hypertensive therapy, other serious system diseases (malignant tumor, gastrointestinal hemorrhage, gastric ulcer, and so on) and function examination of liver and kidney (more than 1.5 times the reference value of aspartate aminotransferase (AST), alanine transaminase (ALT), creatinine (Cr))
6. Mental disorder
7. Pregnancy, planned pregnancy and breast-feeding women
8. Chinese medicine granule and iodine allergy
9. Glaucoma patients
10. A history of another clinical trial in the previous 2 months
11. No consent form signed

### Handling of withdrawal and dropout

1. Voluntarily withdrawal
2. Loss of follow-up
3. Poor compliance and presence of severe adverse effects
4. Revealing and uncovering blind in urgency
5. Misdiagnosis
6. Using forbidden drugs or treatments in the course of the trial
7. Taking no medication during the trial
8. No evaluable records after medication

Reasons for withdrawing participants will be recorded in CRFs, and the last data would be included in data analysis.

The whole research plan would be terminated for the following circumstances:

1. Occurrence of serious adverse events related to the research medication
2. Flawed protocol
3. Funding and management problem due to the sponsors
4. Administrative authorities terminate the trial

### Recruitment strategies

We will apply two strategies to recruit participants with angina pectoris. Firstly, we will recruit participants from out-patient clinics and in-patient department in every clinical center. Secondly, potential patients in local communities and out-hospital clinics will be recruited by advertisements in the post, leaflets, TV broadcast and newspapers. The posters will contain brief introductions about the population needed to be involved, the free treatments offered to eligible participants and the contact information of the researcher. Participants will be included if they meet the inclusion criteria and have provided written informed consent indicating that they agree to this trial and join in the trial voluntarily.

### Intervention

#### TCM intervention

Participants randomized to treatment Group 1 will take *Yi-Qi-Jian-Pi* and *Qu-Tan-Hua-Zhuo* granule, which is composed of *HuangQi* (*Astragalus membranaceus*) - 10 g, *DangShen* (*Codonopsis pilosula*) - 10 g, *BaiZhu* (*Atractylodes macrocephala Koidz*) - 10 g, *FuLing* (*Poria cocos Wolf*) - 10 g, *QingBanXia* (*Rhizoma pinelliae Preparata*) - 6 g, *GuaLou* (*Trichosanthes kirilowii Maxim*) - 10 g, *DanShen* (*Salvia miltiorrhiza*) - 10 g, *ZhiGanCao* (*Glycyrrhiza uralensis Fisch*) - 3 g. Those in treatment Group 2 (*Yi-Qi-Jian-Pi* and *Qu-Tan-Hua-Yu* granule) are composed of *DanShen* (*Salvia miltiorrhiza*) - 10 g, *BaiZhu* (*Atractylodes macrocephala Koidz*) - 10 g, *FuLing* (*Poria cocos Wolf*) - 10 g, *ZhiGanCao* (*Glycyrrhiza uralensis Fisch*) - 3 g, *QingBanXia* (*Rhizoma pinelliae Preparata*) - 6 g, *GuaLou* (T*richosanthes kirilowii Maxim*) - 10 g, *TaoReng* (*Prunus persica Batsch*) - 10 g, *HongHua* (*Carthamus tinctorius*) - 6 g. The action and batch number of each herb is summarized in Table 1[7]. The control group is made from *Yi-Qi-Jian-Pi* and *Qu-Tan-Hua-Zhuo* granules (10%) and starch (90%) to achieve the same shape, color, smell, taste, texture package and Lot Number. Patients are instructed to dissolve granules into 100 ml of hot water and to take the solution orally between 30°C to 37°C twice daily for 12 weeks. Each granule is prepared by Jiangyin Tianjiang Pharmaceuticals Co. Ltd, Jiangsu, China according to the standards of Good Manufactory Practice (GMP) [18].

**Table 1:** **The action and batch number of each herb**

Ingredients	Lot number	Action
*HuangQi* (*Astragalus membranaceus*)	1302026	TCM: tonifying spleen and protecting defensive *Qi*.
		Pharmaceutical study: 1. Promoting metabolism effect. 2. Diuresis effect. 3. Cardiovascular system protecting effect.
*DangShen* (*Codonopsis pilosula*)	1302095	TCM: tonifying *Qi* of spleen and lung and benefiting blood and body fluid.
		Pharmaceutical study: 1. Adjusting gastrointestinal movement effect. 2. Anti-ulcer effect. 3. Immune-enhancing effect. 4. Anti-aging effect.
*BaiZhu* (*Atractylodes macrocephala Koidz*)	1302011	TCM: tonifying *Qi* of spleen, eliminating dampness and diuresis.
		Pharmaceutical study: 1. Intestinal tube two-way regulation effect. 2. Anti-ulcer effect. 3. Diuresis effect. 4. Strong effect.
*FuLing* (*Poria cocos Wolf*)	1301056	TCM: diuresis, detumescence, tonifying *Qi* of spleen and tranquilization.
		Pharmaceutical study: 1. Diuresis effect. 2. Calming effect. 3. Anti-tumor effect. 4. Anti-diabetic effect. 5. Enhance myocardial contractility.
*QingBanXia* (*Rhizoma pinelliae Preparata*)	1301078	TCM: eliminating dampness, resolving phlegm, controlling vomiting and eliminating stagnation.
		Pharmaceutical study: 1. Vomiting control effect. 2. Anti-tumor effect. 3. Gastric secretion inhibition effect.
*GuaLou* (*Trichosanthes kirilowii Maxim*)	1301157	TCM: clearing heat, reducing phlegm, eliminating stagnation and relaxing bowels.
		Pharmaceutical study: 1. Reducing phlegm effect. 2. Coronary artery dilatation effect. 3. Lipid-lowering effect.
*DanShen* (*Salvia miltiorrhiza*)	1301173	TCM: activating and cooling blood, regulating menstruation, removing stasis and pain.
		Pharmaceutical study: 1. Improving myocardial ischemia effect. 2. Improving microcirculation effect. 3. Hypotensive effect. 4. Platelet aggregation inhibition effect. 5. Liver protection effect. 6. Anti-inflammatory effect.
*ZhiGanCao* (*Glycyrrhiza uralensis Fisch*)	1212045	TCM: tonifying *Qi* of spleen, eliminating phlegm, anti-cough, heat-clearing and detoxifying and reconciling.
		Pharmaceutical study: 1. Anti-arrhythmia effect.2. Anti-ulcer effect.3.Diuresis effect. 4. Analgesia effect. 5. Anti-tussive effect. 6. Eliminating phlegm effect.
*TaoReng* (*Prunus persica Batsch*)	1212013	TCM: activating blood, removing stasis, relaxing bowels and relieving cough and asthma.
		Pharmaceutical study: 1. Cerebral blood flow improving effect. 2. Anti-coagulant effect. 3. Analgesia effect. 4. Anti-allergic effect. 5. Relieving cough and asthma effect.
*HongHua* (*Carthamus tinctorius*)	1212080	TCM: activating blood, regulating menstruation, removing stasis and pain.
		Pharmaceutical study: 1. Improving myocardial ischemia and coronary artery flow effect. 2. Anti-arrhythmia effect. 3. Hypotensive effect. 4. Platelet aggregation inhibition effect. 5. Analgesia and sedation effect. 6. Anti-inflammatory effect.

#### WM conventional intervention

An anti-anginal drug, nitroglycerin, is selected as WM conventional treatment and allowed to be taken at a dose of 0.5 g sub-lingual at at least 5-minute intervals [19].

### Concomitant treatments and forbidden drugs

1. Patients are not allowed to take any other TCM intervention (acupuncture treatment, angina pectoris plaster, and so on), nitrates except for nitroglycerin, non-dihydropyridine CCBs and lipid-lowering drugs
2. In the run-in and study period, usage of aspirin, BBs and dihydropyridine CCBs in the previous 4 weeks is allowed to continue and conversion is prohibited
3. The dosage, duration and name of any concomitant treatment or medication must be recorded carefully in the CRFs

### Drug management

Seventy-five to 120% of drug usages are eligible for protocol plan. The three groups’ granules will be prepared respectively but uniformly packaged. The package, drug name, function and indication, usage and dosage, storage condition, valid period and name of the manufacturer will be marked and a tag indicating ‘trial use’ will be attached. Drugs must be kept in the appropriate temperature in a dry, shady and cool place. Drug administrators should return unused drugs to estimate participant compliance and note these in the CRFs.

### Outcomes

#### Efficacy outcomes

The primary outcomes include:

1. Frequency of angina pectoris attacks
2. Dosage of nitroglycerin
3. Body limited dimension of Seattle Angina Questionnaire (SAQ)

The secondary outcomes include:

1. SAQ except for the body limited dimension
2. TCM pattern questionnaire
3. The duration of angina pectoris
4. Angina pain severity

#### Therapeutic mechanism outcomes

Blood serum metabolites; total cholesterol (TC); triglycerides (TG); low-density lipoprotein (LDL); high-density lipoprotein (HDL); ox-LDL; hypersensitive C reactive protein; homocysteine; glucagon like peptide-1; angiotonin II; HMG-COA reductase; adherence factor PS; scavenger receptor A; scavenger receptor B; chemokine MCP-1; adherence factor vascular cell adhesion protein (VCAM)-1; 5-HT; vasointestinal peptide (VIP); ghrelin; norepinephrine (NE); acetylcholine (Ach); mtDNA copy number; MicroRNA155; MicroRNA 33; MicroRNA 126; MicroRNA 125; MicroRNA 221; MicroRNA 222.

#### Safety outcomes

For the sake of participant safety, the following biological indicators will be monitored and watched throughout the trial: blood routine, liver function (AST, ALT), renal function (BUN, Cr), and electrocardiograph (ECG). Adverse events are defined as any unexpected or uncomfortable signs, symptoms, or diseases. Once adverse events happen during the treatment and follow-up period, all the details should be documented in the CRF form and reported to the ethics committee immediately so that they can make a decision about whether the patient should withdraw from the trial.

#### Endpoint outcomes

Death incident: cardiovascular death, cerebrovascular death, sudden death, other cause of death.

Cardiovascular incident: unstable angina pectoris hospitalization, acute myocardial infarction, malignant arrhythmia, cardiac shock, revascularization and other cardiovascular incident.

Non-cardiovascular incident: stroke, pulmonary embolism, peripheral vessel, tumor, other non-cardiovascular incident.

All three groups will undergo a twelve-week treatment and two-week follow-up. Four visits in sum will be scheduled for each subject: one visit each in week 0, week 4, week 12 and week 14. The CRFs contains a range of information and this will be completed by the corresponding researchers at each center. Trial coordinators and therapists at each center will record above endpoint outcome and the details of these events throughout the treatment and follow-up period. Outcomes to be measured and the time points of data collection can be found in Additional file 1: Table S1.

### Quality control

Quality control will be conducted by rigorous monitoring throughout the trial. All staff who enroll participants, assessors who collect data and management who distribute drugs must attend training to make sure all practices at each hospital are generalized and standardized according to the standard operating procedures (SOP). Physicians must pass the required training test to understand the purpose and content of the trial, intervention strategies and any adverse events observation. To guarantee compliance, researchers must remind the participants of the regular visits ahead of 2 or 3 days.

### Sample size calculation

According to the response rates of treatment, Group 1 and placebo were 88.5% and 60%, respectively; 37 patients of each group was estimated as sufficient to achieve 80% power in detecting treatment differences, based on two-sided Chi-square test without continuity correction at a significance level of 0.05 (α = 0.05; 1-β = 0.80). In the same way, assuming the clinical effective rates of Group 2 and placebo were 82.5% and 60%, respectively and 64 patients of each group was estimated as sufficient to achieve 80% power. Meanwhile, taking into account a dropout of 15%, we concluded that a total of 240 patients with 80 for each group would need to be recruited to ensure statistically significant results.

### Statistical analysis

The statistical analysis will be conducted by a statistician of the Beijing Bozhiyin Science and Technology Co. Ltd, China. They are blinded to the whole trial and using Statistical Analysis System (SAS, Cary, NC, USA) version 9.2 and Statistic Package for Social Science (SPSS, Chicago, IL, USA) version 17.0 software packages. All mechanism, efficacy and safety analyses will be strictly conducted according to the intention-to-treat (ITT) principle and the full analysis set (FAS) population, the per-protocol set (PPS) population and the safety set (SS) population. Missing values will be replaced by the last observation carried forward (LOCF) method.

The statistical significance is defined as a two-sided *P*-value of <0.05 and 95% confidence interval. For descriptive statistics, continuous variables will be described as numerous, mean ± standard deviation, median, Q1 to Q3 and mini-maxi and dichotomous data will be reported as frequency and percentage. Baseline differences among the groups will be assessed with the use of Student’s *t*-test for normally distributed continuous variables and the non-parametric Mann-Whitney *U*-test for non-normally distributed variables. Comparisons between groups will be conducted by using an analysis of covariance (ANCOVA) with baseline as covariate. For efficacy analysis, changes from baseline to endpoint of treatment in scores will be tested with repeated measure analysis of variance (ANOVA). Within group differences will be assessed with paired *t*-test for normally distributed data and Wilcoxon signed-rank test for non-normally distributed data. Mechanism will be assessed between mechanism index and primary outcomes by ANCOVA and regression analysis. Safety will be assessed by participant compliance and adverse events. The endpoint events will be listed by detailed account.

### Ethics issue and informed consent

This trial is in accordance with the principles of the Declaration of Helsinki (version Edinburgh 2000). The study protocol was reviewed and approved by IRB of the Affiliated Hospital of Liaoning University of TCM (Number 2013CS (KT)-002-01), IRB of the Liaoning Institute of Chinese Medicine (Number 20130509-1), IRB of the Affiliated Hospital of Inner Mongolia National University, IRB of the Shen-Zhou Hospital of Shenyang Medical College, IRB of the First Affiliated Hospital of China Medical University and IRB of the Sheng-Jing Hospital of China Medical University. Before randomization, all eligible patients are informed of the details of the study and all the benefits and risk that they may take from this trial. Meanwhile, we require all patients to sign the written informed consent prior to enrollment, and they are given enough time to decide whether they will take part in this trial.

## Discussion

SAP is a major public health problem, which can endanger a patient’s life quality. Currently, TCM has a considerable role in the symptom and quality of life improvement of SAP patients in China and worldwide.

Our research team has conducted a deep study of the pathogenesis, TCM pattern feature and efficacy evaluation of TCM in SAP. We proposed that the key pathogenic mechanism was phlegm and blood stasis integration owing to spleen deficiency generating phlegm and phlegm leading to blood stasis [20]. Otherwise, we have developed pattern differentiation standard of SAP and TCM pattern diagnosis and efficacy evaluation scale for pattern of phlegm and blood stasis integration [21, 22].

To our knowledge, this may be the first trial to utilize high-quality EBM evidence to assess the effectiveness and safety of SAP via TCM pattern differentiation, as well as exploring the efficacy mechanism and regulation with the molecular biology and systems biology. In the present study, we establish two treatment groups and one control group in this trial. Our aim of establishing these three groups is to differentiate and isolate the placebo effect and research bias; in particular, to further appropriately validate the accumulation effect of activating blood and removing stasis on the basis of tonifying spleen and eliminating phlegm. It is worth mentioning that we tried to contact the central regulation with the effect organ through multiple communication pathways, such as brain-gut axis regulation of lipid metabolism, immune inflammation, and vascular endothelial network, so as to explain the specific process of the efficacy mechanism and regulation of SAP from spleen.

We have presented the design and protocol for a randomized controlled trial of patients with SAP together with spleen deficiency and phlegm pattern. The clinical trial protocol plays a vital role of study conduct, reporting, and appraisal. To facilitate appropriate high quality methodology and strict quality control, this protocol has been developed according to the CONSORT statement, SPIRIT 2013, and SPIRIT 2013 explanation and elaboration [23]. We particularly described our method of recruitment, randomization and allocation concealment, and data collection. In addition, we reported the TCM intervention according to Recommendations for Reporting Randomized Controlled Trials of Herbal Interventions, including dosage, action, batch number, and quantitative description. Completion of this trial may highlight evidence on the effectiveness and safety of TCM and provide the approaches on the efficacy mechanism and regulation of TCM pattern. The results of this study may generate scientific and rigorous evidence for the study of SAP via TCM pattern differentiation.

## Trial status

At the time of manuscript submission, patient recruitment for the trial is on-going.

## Electronic supplementary material

Additional file 1: Table S1: Schedule of enrollment, intervention and assessments. (PDF 40 KB)

Below are the links to the authors’ original submitted files for images.

Authors’ original file for figure 1

Authors’ original file for figure 2

## Abbreviations

Ach: acetylcholine
ALT: alanine transaminase
ANCOVA: analysis of covariance
ANOVA: analysis of variance
AST: aspartate aminotransferase
BBs: β blockers
BUN: blood urea and nitrogen
CCBs: calcium channel blockers
CCS: Canadian Cardiovascular Society
CHD: coronary heart disease
CONSORT: Consolidated Standards of Reporting Trials
Cr: creatinine
CRFs: Case Report Forms
CT: computed tomography
EBM: evidence-based medicine
ECG: electrocardiograph
FAS: full analysis set
GERD: gastric and esophageal reflux disease
GMP: Good Manufactory Practice
Group 1: *Yi-Qi-Jian-Pi* and *Qu-Tan-Hua-Zhuo* group
Group 2: *Yi-Qi-Jian-Pi* and *Qu-Tan-Hua-Yu* group
HCM: hypertrophic cardiomyopathy
HDL: high-density lipoprotein
HTS: high throughput screening
IRB: Institutional Review Board
ITT: intention-to-treat
LAD: left anterior descending
LCX: left circumflex
LDL: low-density lipoprotein
LM: left main stem
LOCF: last observation carried forward
NE: norepinephrine
PPS: per-protocol set
RCA: right coronary artery
SAP: stable angina pectoris
SAQ: Seattle Angina Questionnaire
SAS: Statistical Analysis System
SOP: standard operating procedures
SPSS: Statistic Package for Social Science
SS: safety set
TC: total cholesterol
TCM: traditional Chinese medicine
TG: triglycerides
UAP: unstable angina pectoris
VCAM-1: vascular cell adhesion protein-1
VIP: vasointestinal peptide
WHO: World Health Organization
WM: western medicine.
