# Supplementary material for: Traditional Chinese medicine for stable angina pectoris via TCM pattern differentiation and TCM mechanism: study protocol of a randomized controlled trial
Source: Trials. 2014 Oct 30;15:422. doi: 10.1186/1745-6215-15-422 (PMC4233055; doi:10.1186/1745-6215-15-422)
Supplement: Supplementary file 1 — Additional file 1: Table S1: Schedule of enrollment, intervention and assessments. (PDF 40 KB) [file 13063_2014_2290_MOESM1_ESM.pdf]

Table 2 Schedule of enrollment, intervention and assessments

TIMEPOINT※	Enrollment	Run-in	Allocation	Treatment		Follow-ups
	-2	-2 - 0	0	4	12	14
**ENROLMENT:**						
Eligibility screen	×					
Informed consent	×					
Demographic information	×					
Blood test			×		×	
Electrocardiogram			×	×	×	
Disease, Treatment, Allergy history	×		×	×	×	
Allocation			×			
Combined medication			×	×	×	
Randomization			×			
Drug distribution			×	×		
Drug reclaiming records			Record each trail medicine reclaiming			
**INTERVENTION:**						
Group 1（Yi-Qi-Jian-Pi and Qu- Tan-Hua-Zhuo)			◆	─	◆	
Group 2(Yi-Qi-Jian-Pi and Qu- Tan-Hua-Yu)			◆	─	◆	
Placebo			◆	─	◆	
**ASSESSMENTS:**						
Primary outcome			○	○	○	
Secondary outcome			○	○	○	
Endpoint incident			○	○	○	○
Therapeutic mechanism			○	○	○	
Patient’s compliance			×	×	×	
Adverse reaction			×	×	×	×
Serious adverse events			×	×	×	×
Safety assessment			×			
Research conclusion			×			
Statistic analysis			○			

“×”:Trail management board; “○”:Research center management;

※According to SPIRIT 2013 Statement: Defining Standard Protocol Items for Clinical Trials
