# Supplementary figures and images for: Traditional Chinese medicine for stable angina pectoris via TCM pattern differentiation and TCM mechanism: study protocol of a randomized controlled trial
Source: Trials. 2014 Oct 30;15:422. doi: 10.1186/1745-6215-15-422 (PMC4233055; doi:10.1186/1745-6215-15-422)

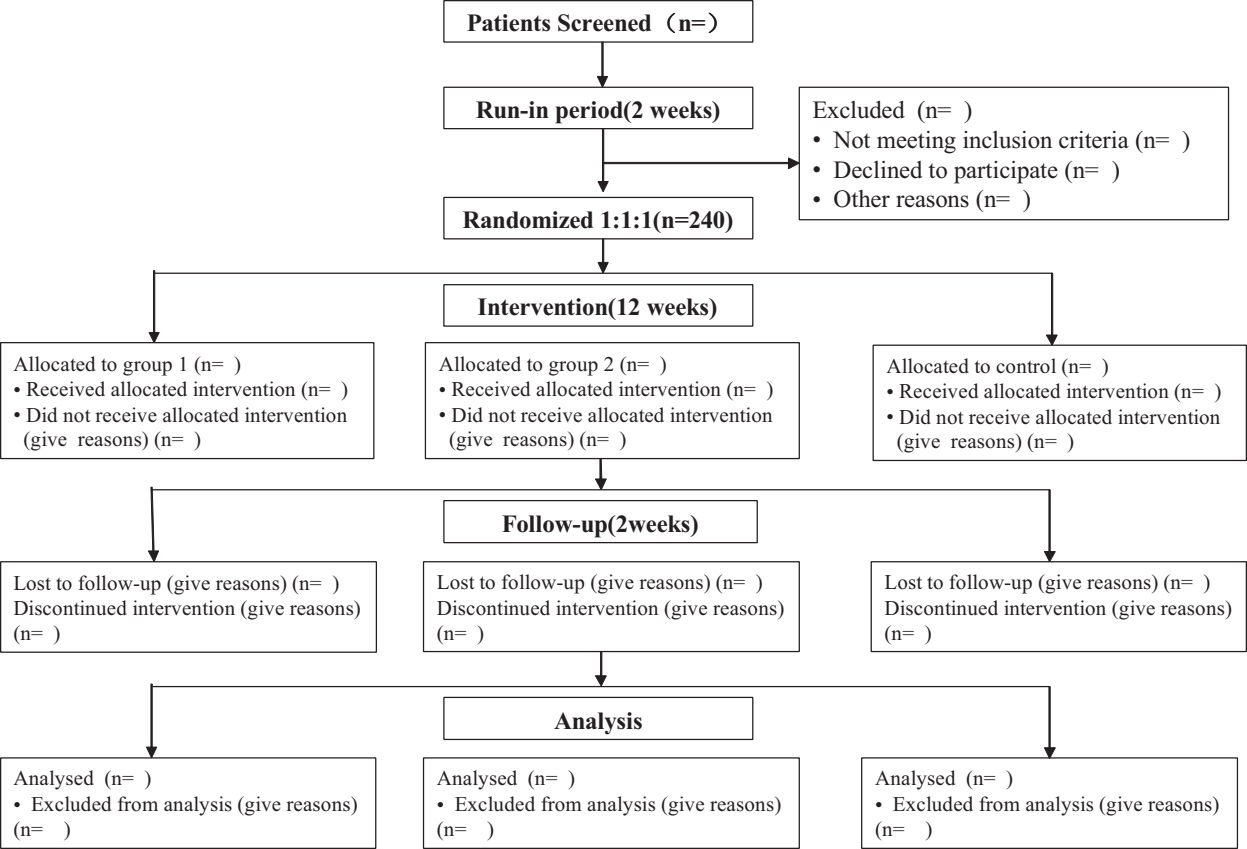

Supplement: Supplementary file 2 — Authors’ original file for figure 1 [file 13063_2014_2290_MOESM2_ESM.pdf]

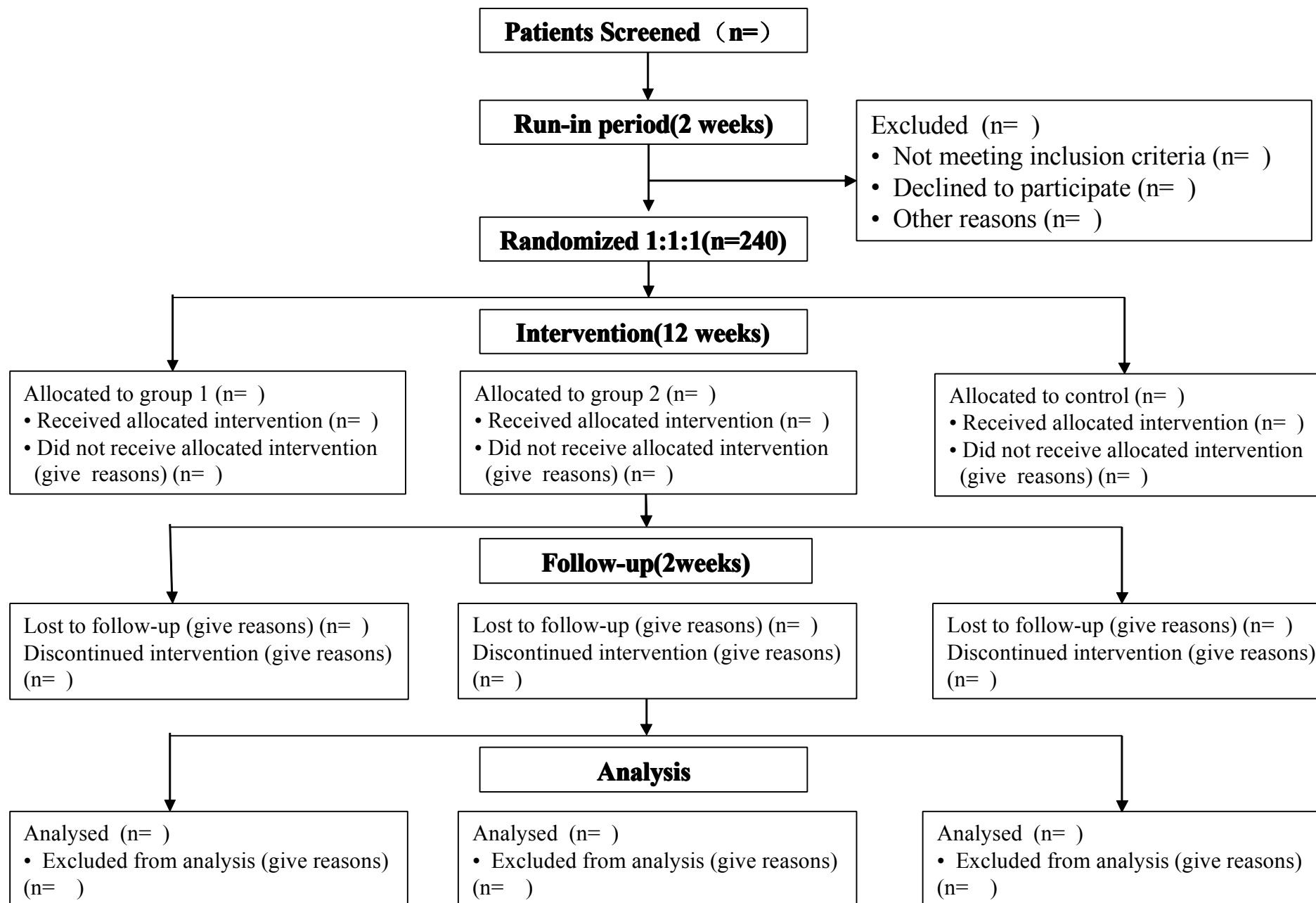

**Figure 1 Trial flow chart**

Supplement: Supplementary file 3 — Authors’ original file for figure 2 [file 13063_2014_2290_MOESM3_ESM.pdf]
